# A11 TELOCYTES-FOXL1+ REGULATES THE EXTRACELLULAR MATRIX NETWORK IN COLITIS-ASSOCIATED CANCER

**DOI:** 10.1093/jcag/gwad061.011

**Published:** 2024-02-14

**Authors:** V Reyes Nicolas, A B Alfonso, J Raisch, F Boisvert, M A Lauzon, N Perreault

**Affiliations:** IBC, Universite de Sherbrooke Faculte de Medecine et des Sciences de la Sante, Sherbrooke, QC, Canada; IBC, Universite de Sherbrooke Faculte de Medecine et des Sciences de la Sante, Sherbrooke, QC, Canada; IBC, Universite de Sherbrooke Faculte de Medecine et des Sciences de la Sante, Sherbrooke, QC, Canada; IBC, Universite de Sherbrooke Faculte de Medecine et des Sciences de la Sante, Sherbrooke, QC, Canada; Universite de Sherbrooke Faculte de Genie, Sherbrooke, QC, Canada; IBC, Universite de Sherbrooke Faculte de Medecine et des Sciences de la Sante, Sherbrooke, QC, Canada

## Abstract

NOT PUBLISHED AT AUTHOR’S REQUEST

Please acknowledge all funding agencies listed below

Funding Agencies

CIHRDoctoral Scholarship Fonds de recherche du Québec (FRQS)

